# Risk factors and incidence of third trimester stillbirths in China

**DOI:** 10.1038/s41598-021-92106-1

**Published:** 2021-06-16

**Authors:** Ying Hu, Qi Wu, Jia Liu, Die Hong, Yuqing Zou, Jingjing Lu, Yuhui Wang, Danqing Chen, Lu Qi, Zhaoxia Liang

**Affiliations:** 1 Obstetrical Department, Women’s Hospital, Zhejiang University School of Medicine, Hangzhou, 310006 China; 2 Department of Epidemiology, School of Public Health and Tropical Medicine, Tulane University, New Orleans, LA USA

**Keywords:** Pregnancy outcome, Risk factors

## Abstract

About 2.6 million third-trimester stillbirths occur annually worldwide, mostly in low- and middle-income countries. However, the causes of stillbirths are rarely investigated. We performed a retrospective, hospital-based study in Zhejiang Province, southern China, of the causes of third-trimester stillbirths. Causes of stillbirths were classified using the Relevant Condition at Death classification system. From January 1, 2013, to December 31, 2018, we enrolled 341 stillbirths (born to 338 women) from 111,275 perinatal fetuses (born to 107,142 women), as well as 293 control cases (born to 291 women). The total incidence of third-trimester stillbirths was 3.06/1000 (341/111,275). There were higher proportions of women with a high body mass index, twins, pregnancy-induced hypertension, assisted reproduction and other risk factors among the antepartum than the control cases. The antepartum stillbirth fetuses were of lower median birth weight and gestational age and had a smaller portion of translucent amniotic fluid than the control cases. The antepartum stillbirth fetuses had a higher frequency of abnormalities detected prenatally and of fetal growth restriction than the control cases. Of 341 cases (born to 338 mothers), the most common causes of stillbirth were fetal conditions [117 (34.3%) cases], umbilical cord [88 (25.8%)], maternal conditions [34 (10.0%)], placental conditions [31 (9.1%)], and intrapartum [28 (8.2%)]. Only eight (2.3%), three (0.9%), and two (0.6%) stillbirths were attributed to amniotic fluid, trauma, and uterus, respectively. In 30 (8.8%) cases, the cause of death was unclassified. In conclusion, targeted investigation can ascertain the causes of most cases of third-trimester stillbirths.

## Introduction

Stillbirth refers to intrauterine death of the fetus during pregnancy or parturition, who was delivered without any vital signs. The rate of stillbirths is an important indicator of the obstetric quality and comprehensive medical strength of a country or region. Although the stillbirth rate decreased from 2000 to 2015, there were 4.8 million perinatal deaths worldwide in 2015, of which 2.6 million were third-trimester stillbirths and 98% occurred in low- and middle-income countries (LMICs). It was reported that 36% of third-trimester stillbirths occurred in south-Asian LMICs, and 41% in African LMICs^1,2^. More importantly, stillbirths have marked socioeconomic consequences, including impairment of the physical and mental health of the parents and the financial cost to family and to the healthcare system^3^. Therefore, the World Health Assembly-endorsed Every Newborn Action Plan acknowledged the need to reduce the number of stillbirths in LMICs, with a target of reducing the number of stillbirths from 18.4 per 1000 births in 2015 to 12 per 1000 births by 2030^4^. As one of the largest LMICs, the total incidence of stillbirths in China is 1–4%, and its number of stillbirths is in the top five worldwide^5,6^. Therefore, China must improve monitoring and reduce the number of preventable stillbirths.

The most effective means of reducing the incidence of stillbirths is to identify risk factors and provide targeted treatment. Pregnancy complications, such as pre-eclampsia and placental abruption, can lead to stillbirth, as can maternal infection^7^. However, 25–60% of stillbirths are unexplained^8^, possibly because of inadequate evaluation^9^. A systematic review of stillbirths from 2009 to 2016 in LMICs and high-income countries highlighted the poor quality of the data^10^, which hampers the development of strategies to reduce the incidence of stillbirths. The use of 34 classification systems for the causes of stillbirth undermines our understanding of stillbirth epidemiology. In addition, placental examination was performed, in some cases, in two studies from middle-income countries, but not in 28 studies from low-income countries; that method is useful for identifying risk factors^11,12^. Few high-quality biological investigations of the risk factors for stillbirths in LMICs have been performed.

The aim of this retrospective study was to investigate the incidence of third-trimester stillbirths and the risk factors thereof among women who attended the Women’s Hospital, Zhejiang University School of Medicine, China, from 2013 to 2018. We wish to provide reliable data that will enable the incidence of preventable stillbirths to be reduced, particularly in LMICs.

## Results

### Incidence of third-trimester stillbirths

**Figure 1:**
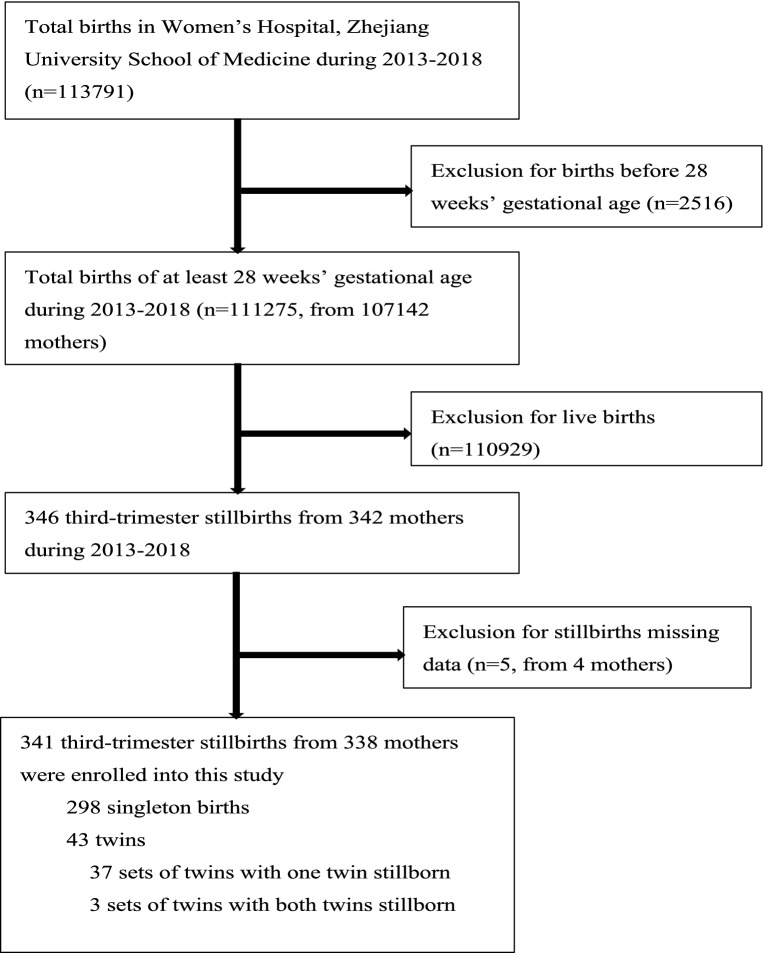
Third-trimester stillbirths in Women’s Hospital, Zhejiang University School of Medicine between Jan 1, 2013 and Dec 31, 2018.

**Figure 2:**
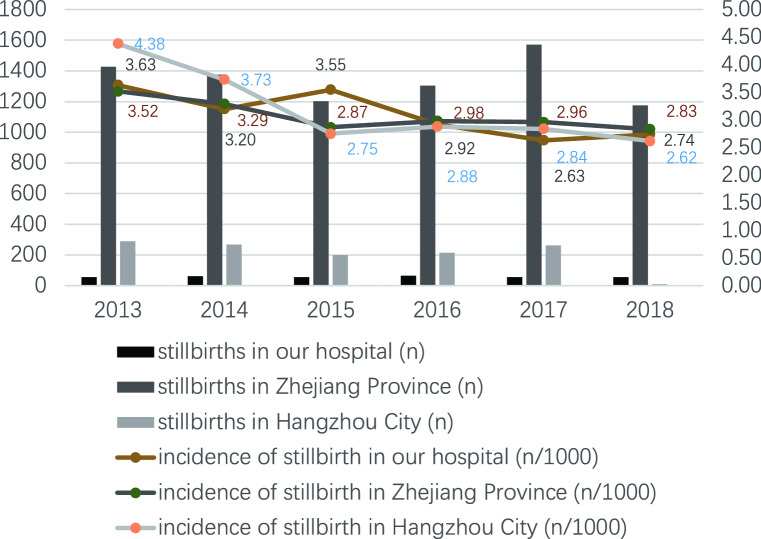
Incidence of third-trimester stillbirths in different years.

**Figure 3:**
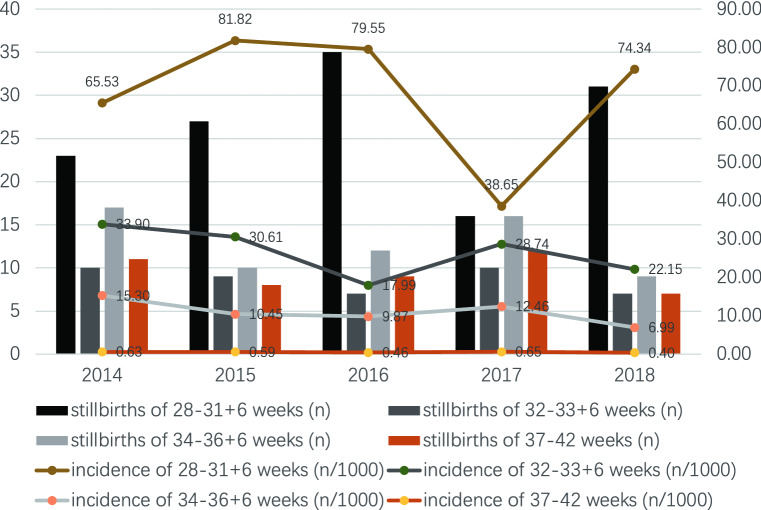
Incidence of third-trimester stillbirths in different years with different gestational age.

From January 1, 2013, to December 31, 2018, the total number of perinatal fetuses delivered in the Women’s Hospital, Zhejiang University School of Medicine was 111,275 (from 107,142 mothers), of which 341 were third-trimester stillbirths from 338 mothers (Fig. 1). The incidence of third-trimester stillbirths was 3.06/1000 (341/111,275). The lowest incidence was in 2017 (2.63/1000, 54/20,533) and the highest was in 2013 (3.63/1000, 55/15,134) (Fig. 2). The incidence of third-trimester stillbirths was 3.07/1000 in Zhejiang Province and 3.16/1000 in Hangzhou City. Figure 2 also presented the specific incidence from 2013 to 2018. The overall incidence of third-trimester stillbirths decreased with increasing gestational age in the previous 5 years: the highest incidence was 81.82/1000 (27/330) at 28–31 + 6 weeks of gestational age in 2015, and the lowest was 0.4/1000 (7/17,675) at 37–42 weeks of gestation in 2018 (Fig. 3). Moreover, the incidence of third-trimester stillbirths varied according to demographic features (Supplement: Incidence of third-trimester stillbirths in women and fetuses with different demographic features). These features may be risk factors for stillbirth (Table S1). We next analyzed 341 third-trimester stillbirths (338 mothers) and the corresponding controls.

### Baseline demographic and clinical features of women who had stillbirths

Of the 338 women who had third-trimester stillbirths from January 1, 2013, to December 31, 2018, 310 were categorized as antepartum cases and the remaining 28 as intrapartum cases; 291 control cases were also analyzed. There were higher proportions of women with a high body mass index (BMI), minority nationality, primary education level, unemployed occupation, and previous inevitable abortion among the antepartum than the control cases. Only three (0.9%) of 338 women had a history of stillbirth (Table 1).

**Table 1:** Demographic and baseline clinical features of women who had stillbirths.

Baseline features of women	Total women of stillbirths (n = 338)	Intrapartum (n = 28)	Antepartum (n = 310)	Control (n = 291)	*P* value*
**Age**					0.557
< 20	4/338 (1.2%)	2/28 (7.1%)	2/310 (0.6%)	1/291 (0.3%)	
20–29	165/338 (48.8%)	9/28 (32.1%)	156/310 (50.3%)	141/291 (48.5%)	
30–34	108/338 (32.0%)	9/28 (32.1%)	99/310 (31.9%)	107/291 (36.8%)	
≥ 35	61/338 (18.0%)	8/28 (28.6%)	53/310 (17.1%)	42/291 (14.4%)	
**BMI**					0.003
< 18.5	1/338 (0.3%)	0	1/310 (0.3%)	0	
18.5 ≪ BMI ≪ 25	148/338 (43.8%)	10/28 (35.7%)	138/310 (44.5%)	101/291 (34.7%)	
25 < BMI < 28	103/338 (30.5%)	10/28 (35.7%)	93/310 (30.0%)	129/291 (44.3%)	
≫ 28	86/338 (25.4%)	8/28 (28.6%)	78/310 (25.2%)	61/291 (21.0%)	
**Nationality**					0.041
Han	327/338 (96.7%)	28/28 (100%)	299/310 (96.5%)	288/291 (99.0%)	
Minority	11/338 (3.3%)	0	11/310 (3.5%)	3/291 (1.0%)	
**Education level**					0.000
Primary	21/338 (6.2%)	6/28 (21.4%)	15/310 (4.8%)	2/291 (0.7%)	
Secondary	119/338 (35.2%)	14/28 (50.0%)	105/310 (33.9%)	63/291 (21.7%)	
College	177/338 (52.4%)	8/28 (28.6%)	169/310 (54.5%)	200/291 (69.0%)	
Post-graduate	21/338 (6.2%)	0	21/310 (6.8%)	25/291 (8.6%)	
**Occupation**					0.000
Staff	211/338 (62.4%)	9/28 (32.1%)	202/310 (65.2%)	258/291 (88.7%)	
Peasant	14/338 (4.1%)	3/28 (10.7%)	11/310 (3.5%)	4/291 (1.4%)	
Individual enterprise	29/338 (8.6%)	3/28 (10.7%)	26/310 (8.4%)	11/291 (3.8%)	
Unemployed	84/338 (24.9%)	13/28 (46.4%)	71/310 (22.9%)	18/291 (6.2%)	
**Gravidity (before current pregnancy)**					0.162
0	124/338 (36.7%)	8/28 (28.6%)	116/310 (37.4%)	127/291 (43.6%)	
1–4	194/338 (45.9%)	9/28 (32.1%)	185/310 (59.7%)	160/291 (55.0%)	
≫ 5	20/338 (17.5%)	11/28 (39.3%)	9/310 (2.9%)	4/291 (1.4%)	
**Parity (before current pregnancy)**					0.550
0	211/338 (62.4%)	13/28 (46.4%)	198/310 (63.9%)	179/291 (61.5%)	
≫ 1	127/338 (37.6%)	15/28 (53.6%)	112/310 (36.1%)	112/291 (38.5%)	
**Maternal basic medical condition**					
Hepatitis B	23/338 (6.8%)	0	23/310 (7.4%)	4/291 (1.4%)	0.000
Uterine malformation	9/338 (2.7%)	0	9/310 (2.9%)	0	0.004
Chronic hypertensive disorder	8/338 (2.4%)	3/28 (10.7%)	5/310 (1.6%)	0	0.062
Hypothyroidism	5/338 (1.5%)	0	5/310 (1.6%)	6/291 (2.1%)	0.682
Syphilis	5/338 (1.5%)	1/28 (3.6%)	4/310 (1.3%)	0	0.250
Congenital heart disease	3/338 (0.9%)	1/28 (3.6%)	2/310 (0.6%)	1/291 (0.3%)	1.000
Diabetes	3/338 (0.9%)	0	3/310 (0.6%)	2/291 (0.7%)	1.000
Psychiatric disorders	2/338 (0.6%)	0	2/310 (0.6%)	0	0.500
Other basic medical conditions	15/338 (4.4%)	0	15/310 (4.8%)	9/291 (3.1%)	0.275
** Previous uterine operation**	77/338 (22.8%)	2/28 (7.1%)	75/310 (24.2%)	60/291 (20.6%)	0.294
**Previous inevitable abortion**					0.001
0	280/338 (82.8%)	22/28 (78.6%)	258/310 (83.2%)	284/291 (97.6%)	
1	46/338 (13.6%)	5/28 (17.9%)	41/310 (13.2%)	5/291 (1.7%)	
≫ 2	12/338 (3.6%)	1/28 (3.6%)	11/310 (3.5%)	2/291 (0.7%)	
** Previous stillbirths**	3/338 (0.9%)	0	3/310 (1.0%)	3/291 (1.0%)	0.938
**ABO blood group**					0.808
A type	96/338 (28.4%)	9/28 (32.1%)	87/310 (28.1%)	78/291 (26.8%)	
B type	99/338 (29.3%)	11/28 (40.7%)	88/310 (28.4%)	75/291 (25.8%)	
O type	111/338 (32.8%)	6/28 (22.2%)	105/310 (33.9%)	108/291 (37.1%)	
AB type	32/338 (9.5%)	2/28 (7.4%)	30/310 (9.7%)	30/291 (10.3%)	
**Rh blood group**					
Positive	338/338 (100%)	28/28 (100%)	310/310 (100%)	291/291 (100%)	
Negative	0	0	0	0	

Data are n/N (%).

*Comparison of antepartum and control cases.

### Clinical features during the current pregnancy of women who had stillbirths

There were higher proportions of women with twins, no antenatal care visit, pregnancy-induced hypertension, assisted reproduction, vaginal delivery, and history of preventing miscarriage among the antepartum than the control cases. Pregnancy-induced hypertension included hypertension (12, 3.6%), preeclampsia (10, 3.0%), and severe preeclampsia (32, 9.5%). Of the 338 women, 168 had abnormal fetal movement before diagnosis of stillbirth (Table 2).

**Table 2:** Clinical features of women who had stillbirths during the current pregnancy.

Clinical features of women	Total women of stillbirths (n = 338)	Intrapartum (n = 28)	Antepartum (n = 310)	Control (n = 291)	*P* value*
**Multiple gestation**					0.000
Singleton	298/338 (88.2%)	25/28 (89.3%)	273/310 (88.1%)	289/291 (99.3%)	
Twins	40/338 (11.8%)	3/28 (10.7%)	37/310 (11.9%)	2/291 (0.7%)	
MCMA	1/338 (0.3%)	0	1/310 (0.3%)		
MCDA	20/338 (6.0%)	2/28 (7.1%)	18/310 (5.8%)	1/291 (0.3%)	
DCDA	17/338 (5.0%)	0	17/310 (5.5%)	1/291 (0.3%)	
Unknown chorionic property	2/338 (0.6%)	1/28 (3.6%)	1/310 (0.3%)		
**Antenatal care visit**					0.000
None	44/338 (13.0%)	12/28 (42.9%)	32/310 (10.3%)	0	
Our hospital	181/338 (53.6%)	11/28 (39.3%)	170/310 (54.8%)	244/291 (83.8%)	
Other hospitals	113/338 (33.4%)	5/28 (17.9%)	108/310 (34.8%)	47/291 (16.2%)	
**Prenatal screening**					
Screening	270/338 (79.9%)	16/28 (57.1%)	254/310 (81.9%)		
Low risk	230/270 (85.2%)	14/16 (87.5%)	216/254 (85.0%)		
Middle risk	18/270 (6.6%)	1/16 (6.3%)	17/254 (6.7%)		
High risk	22/270 (8.1%)	1/16 (6.3%)	21/254 (8.3%)	3/291 (1.0%)	0.225
**Pregnancy complications**					
Pregnancy-induced hypertension (including preeclampsia)	53/338 (15.7%)	6/28	47/310 (15.2%)	5/291 (1.7%)	0.000
ICP	8/338 (2.4%)	0	8/310 (2.6%)	9/291 (3.1%)	0.705
Gestational diabetes mellitus	31/338 (9.2%)	1/28 (3.6%)	30/310 (9.7%)	27/291 (9.3%)	0.867
Thyroid abnormality	36/338 (10.7%)	3/28 (10.7%)	33/310 (10.6%)	17/291 (5.8%)	0.090
**Mode of delivery**					0.000
Vaginal	283/338 (83.7%)	25/28 (89.3%)	258/310 (83.2%)	188/291 (64.6%)	
Caesarean section	55/338 (16.3%)	3/28 (10.7%)	52/310 (16.8%)	103/291 (35.4%)	
**Mode of conception**					0.046
Nature conceived	314/338 (92.9%)	26/28 (92.9%)	288/310 (92.9%)	281/291 (96.6%)	
ART	24/338 (7.1%)	2/28 (7.1%)	22/310 (7.1%)	10/291 (3.4%)	
History of preventing miscarriage	58/338 (17.2%)	3/28 (10.7%)	55/310 (17.7%)	9/291 (3.1%)	0.000
**TORCH**					
Tested	38/338 (11.2%)	0	38/310 (12.2%)		
Positive	1/338 (0.3%)	0	1/310 (0.3%)		
**Parvovirus**					
Tested	14/338 (4.1%)	0	14/310 (4.5%)		
Positive	0/14	0	0		
** Adverse contact in the early pregnancy**	6/338 (1.8%)	1/28 (3.6%)	5/310(1.6%)		
**Clinical manifestation**					
Abnormal fetal movement	168/338 (49.7%)	0	168/310 (54.2%)		
Abnormal imaging examination	66/338 (19.5%)	8/28 (28.6%)	58/310 (18.7%)		
Abdominal pain	25/338 (7.4%)	7/28 (25.0%)	18/310 (5.8%)		
Abnormal fatal heart rate	13/338 (3.8%)	7/28 (25.0%)	6/310 (1.9%)		
Vaginal bleeding	8/338 (2.4%)	2/28 (7.1%)	6/310 (1.9%)		
Abnormal physical examination	8/338 (2.4%)	4/28 (14.3%)	4/310 (1.3%)		
Umbilical cord blood puncture	3/338 (0.9%)	0	3/310 (1.0%)		
**Intrapartum complications**					
Cord prolapse	4/338 (1.2%)	3/28 (10.7%)	1/310 (0.3%)		
Rupture of cord vessels	8/338 (2.4%)	4/28 (14.3%)	4/310 (1.3%)		
Breech delivery	3/338 (0.9%)	3/28 (10.7%)	0		
Fetal distress	1/338 (0.3%)	0	1/310 (0.3%)		
**Placental abnormality** (**clinical)**					
Placenta praevia	17/338 (5.0%)	1/28 (3.6%)	16/310 (5.2%)		
Placental abruption	18/338 (5.3%)	2/28 (7.1%)	16/310 (5.2%)		
Uterine rupture	2/338 (0.6%)	0	2/310 (0.6%)		
**Evidence of maternal infection**	6/338 (1.8%)	0	6/310 (1.9%)		
Temperature ≥ 38 Celsius degrees	3/338 (0.9%)	0	3/310 (1.0%)		
Clinical chorioamnionitis	3/338 (0.9%)	0	3/310 (1.0%)		
Malodorous vaginal discharge	0/338	0	0		
Uterine tenderness	1/338 (0.3%)	0	1/310 (0.3%)		
**Maternal adverse outcome**					
Uteroplacental apoplexy	10/338 (3.0%)	0	10/310 (3.2%)		
Postpartum hemorrhage	13/338 (3.8%)	1/28 (3.6%)	12/310 (3.9%)		
Diffuse intravascular coagulation	7/338 (2.1%)	0	7/310 (2.3%)		
Serious mental disorders	2/338 (0.6%)	0	2/310 (0.6%)		
Hemorrhagic shock	1/338 (0.3%)	1/28 (3.6%)	0		

Data are n/N (%).

*Comparison of antepartum and control cases.

### Baseline demographic and clinical features of the stillbirths

Of the 341 third-trimester stillbirths (338 mothers), 313 were antepartum cases and 28 were intrapartum cases; 327 control cases were also analyzed. The antepartum cases were of lower median birth weight and gestational age and had a lower frequency of translucent amniotic fluid than the control cases. The antepartum cases also had a higher frequency of abnormalities detected prenatally and FGR than the control cases. Among the 341 stillbirths, there were 37 deaths of one twin and six of both twins. Of 298 singleton stillbirths, 97 (32.6%) had FGR (z-score for birthweight for gestational age, <  − 2). Thirty-six stillbirths were autopsied, of which eight had abnormal findings. Eleven stillbirths underwent array chip testing, of which two had abnormal findings (Table 3).

**Table 3:** Baseline demographic and clinical features of the stillbirths.

Clinical features of fetuses	Total stillbirths (n = 341)	Intrapartum (n = 28)	Antepartum (n = 313)	Control (n = 327)	*P* value*
**Sex**					0.808
Male	153/312 (49.0%)	14/26 (53.8%)	139/286 (48.6%)	163/327 (49.8%)	
Female	159/312 (51.0%)	12/26 (46.2%)	147/286 (51.4%)	164/327 (50.2%)	
Unknown	29/341 (8.5%)	2/28 (7.1%)	27/313 (8.6%)	0	
**Birthweight (g)**					0.000
< 500	11/337 (3.3%)	1/28 (3.6%)	10/309 (3.2%)	0	
500–999	54/337 (16.0%)	7/28 (25.0%)	47/309 (15.2%)	1/327 (0.3%)	
1000–1499	83/337 (24.6%)	8/28 (28.6%)	75/309 (24.3%)	4/327 (1.2%)	
1500–2499	115/337 (34.1%)	5/28 (17.9%)	110/309 (35.6%)	34/327 (10.4%)	
2500–4000	68/337 (20.2%)	6/28 (21.4%)	62/309 (20.1%)	278/327 (85.0%)	
> 4000	6/337 (1.8%)	1/28 (3.6%)	5/309 (1.6%)	10/327 (3.1%)	
Unknown	4/341 (1.2%)	0	4/313 (1.3%)	0	
**Gestational age, weeks**					0.000
28 − 31 + 6	152/341 (44.6%)	12/28 (42.9%)	140/313 (44.7%)	26/327 (8.0%)	
32 − 33 + 6	54/341 (15.8%)	4/28 (14.3%)	50/313 (16.0%)	9/327 (2.8%)	
34 − 36 + 6	78/341 (22.9%)	5/28 (17.9%)	73/313 (23.3%)	17/327 (5.2%)	
37–42	57/341 (16.7%)	7/28 (25.0%)	50/313 (16.0%)	275/327 (84.1%)	
**Amniotic fluid color**					0.000
Translucent	40/311 (12.9%)	6/24 (25.0%)	34/287 (11.8%)	281/327 (85.9%)	
Yellow	57/311 (18.3%)	3/24 (12.5%)	54/287 (18.8%)	9/327 (2.8%)	
Brown	170/311 (54.7%)	9/24 (37.5%)	161/287 (56.1%)	11/327 (3.4%)	
Bloody	44/311 (14.1%)	6/24 (25.0%)	38/287 (13.2%)	26/327 (8.0%)	
Unknown	30/341 (8.8%)	4/28 (14.3%)	26/313 (8.3%)	0	
**Fetal abnormality found in prenatal care**					
Structural abnormality	54/341 (15.8%)	5/28 (17.9%)	49/313 (15.7%)	10/327 (3.1%)	0.000
FGR	37/341 (10.9%)	4/28 (14.3%)	33/313 (10.5%)	2/327 (0.6%)	0.000
Others	36/341 (10.6%)	3/28 (10.7%)	33/313 (10.5%)	6/327 (1.8%)	0.000
**Abnormality of umbilical cord found in prenatal care**					
Cord entanglement	4/341 (1.2%)	0	4/313 (1.3%)	29/327 (8.9%)	0.000
Single umbilical artery	13/341 (3.8%)	1/28 (3.6%)	12/313 (3.8%)	1/327 (0.3%)	0.001
Abnormal umbilical blood flow	35/341 (10.3%)	4/28 (14.3%)	31/313 (9.9%)	0	0.000
Others	5/341 (1.5%)	0	5/313 (1.6%)	3/327 (0.9%)	0.496
**Placental abnormality found in prenatal care**					
Placenta praevia	16/341 (4.7%)	1/28 (3.6%)	15/313 (4.8%)	1/327 (0.3%)	0.000
Placental abruption	4/341 (1.2%)	0	4/313 (1.3%)	2/327 (0.6%)	0.442
Abnormal placenta morphology	9/341 (2.6%)	1/28 (3.6%)	8/313 (2.6%)	2/327 (0.6%)	0.058
Others	5/341 (1.5%)	0	5/313 (1.6%)	0	0.028
**Abnormality of amniotic fluid found in prenatal care**					
Polyhydramnios	38/341 (11.1%)	3/28 (10.7%)	35/313 (11.2%)	4/327 (1.2%)	0.000
Oligohydramnios	43/341 (12.6%)	2/28 (7.1%)	41/313 (13.1%)	8/327 (2.4%)	0.000
**Fetal growth restriction of singleton**	97/298 (32.6%)	15/25 (60.0%)	82/273 (30.0%)	1/289 (0.3%)	0.000
− 3 < Z score < − 2	30/298 (10.1%)	5/25 (20.0%)	25/273 (9.2%)	1/289 (0.3%)	
Z score < − 3	67/298 (22.5%)	10/25 (40.0%)	57/273 (20.9%)	0	
**Fetal growth restriction of twin**	27/43 (62.3%)	2/3 (66.7%)	25/40 (62.5%)	1/38 (2.6%)	0.000
− 3 < Z score < − 2	3/43 (7.0%)	0	3/40 (7.5%)	1/38 (2.6%)	
Z score < − 3	24/43 (55.8%)	2/3 (66.7%)	22/40 (55.0%)	0	
**Fetal appearance**					
Fresh	283/341 (83.0%)	28/28 (100%)	255/313 (81.5%)		
Macerated	58/341 (17.0%)	0	58/313 (18.5%)		
**Autopsy**					
Tested	36/341 (10.6%)	0	36/313 (11.5%)		
Abnormal finding	8/36 (22.2%)	0	8/36 (22.2%)		
**Array chip examination**					
Tested	11/341 (3.2%)	0	11/313 (3.5%)		
Abnormal finding	2/11 (18.2%)	0	2/11 (18.2%)		
**Death of twin**	43/341 (12.6%)	3/28 (10.7%)	40/313 (12.8%)		
Death of one twin	37/43 (86.0%)	3/3 (100%)	34/40 (85.0%)		
Twin deaths	6/43 (14.0%)	0	6/40 (15.0%)		

Data are n/N (%).

*Comparison of fetuses of antepartum stillbirths (313 stillbirths from 310 mothers) and control fetuses (the number of control fetuses refers to actual number of live births: the 293 live births from 291 control women plus 34 live births of twins in the antepartum stillbirths).

### Macroscopic and histological placental observations in stillbirths

The cord abnormalities comprised cord knots, cord hyper helix, cord torsion, cord entanglement, cord rupture, rupture of cord vessels, and others. Chorioamnionitis was histologically confirmed in 26 (40.6%) of 64 placentas tested. Among 24 of those cases (24/26, 92.3%) four were grade I, eight were grade II, and 12 were grade III according to the extent of inflammatory cellular infiltration of the placenta (Table 4).

**Table 4:** Macroscopic and histological placental observations in stillbirths.

Macroscopic and histological observations	Total (n = 341)	Antepartum (n = 313)	Intrapartum (n = 28)
**Umbilical cord**			
Cord insertion			
Marginal	7/341 (2.0%)	5/313 (1.6%)	2/28 (7.1%)
Velamentous	11/341 (3.2%)	8/313 (2.6%)	3/28 (10.7%)
Number of cord vessels			
3	335/341 (98.2%)	308/313 (98.4%)	27/28 (96.4%)
2	6/341 (1.8%)	5/313 (1.6%)	1/28 (3.6%)
Cord knots	4/341 (1.2%)	4/313 (1.3%)	0
Cord hyper helix	48/341 (14.1%)	47/313 (15.0%)	1/28 (3.6%)
Torsion of cord	15/341 (4.4%)	12/313 (3.8%)	3/28 (10.7%)
Cord entanglement	38/341 (11.1%)	38/313 (12.1%)	0
Cord rupture	2/341 (0.6%)	2/313 (0.6%)	0
Rupture of cord vessels	8/341 (2.3%)	4/313 (1.3%)	4/28 (14.3%)
Short umbilical cord	5/341 (1.5%)	5/313 (1.6%)	0
Cord entangled by amniotic band	1/341 (0.3%)	1/313 (0.3%)	0
Cord atresia	1/341 (0.3%)	1/313 (0.3%)	0
Cord cyst	1/341 (0.3%)	1/313 (0.3%)	0
Cord congestion	19/341 (5.6%)	19/313 (6.0%)	0
Cord edema	15/341 (4.4%)	15/313 (4.8%)	0
Cord necrosis	5/341 (1.5%)	5/313 (1.6%)	0
Edema degeneration of cord interstitium	1/341 (0.3%)	1/313 (0.3%)	0
**Placenta**			
Chorioamnionitis	26/64 (40.6%)	25/60 (41.7%)	1/4 (25%)
Retroplacental hematoma	4/64 (6.3%)	4/60 (6.7%)	0
Placental infarction	12/64 (18.8%)	11/60 (18.3%)	1/4 (25%)
Intervillous fibrin	14/64 (21.9%)	11/60 (18.3%)	3/4 (75%)
Intervillous thrombi	1/64 (1.6%)	1/60 (1.7%)	0
Villous calcification	25/64 (39.1%)	23/60 (38.3%)	2/4 (50%)
Villous maturation retardation	8/64 (12.5%)	7/60 (11.7%)	1/4 (25%)
Villous necrosis	4/64 (6.35)	4/60 (6.7%)	0
Villous degeneration	2/64 (3.1%)	2/60 (3.3%)	0
Villous hematoma	3/64 (4.7%)	2/60 (3.3%)	1/4 (25%)
Increased of villous syncytial nodules	10/64 (15.6%)	9/60 (15%)	1/4 (25%)
Interstitial villous vessels congestion	1/64 (1.6%)	1/60 (1.7%)	0
Stenosis of villus space	1/64 (1.6%)	1/60 (1.7%)	0

Data are n/N (%).

### Risk factors for third-trimester stillbirth

Using the ReCoDe system, in 311 (91.2%) of 341 cases, the third-trimester stillbirth was attributed to a single underlying condition, whereas no cause was evident in 30 (8.8%) cases (Table 5). Overall, third-trimester stillbirths were most commonly attributed to the fetus [117 (34.3%) cases; 51 (15.0%) with FGR and 42 (12.3%) with lethal congenital anomaly], umbilical cord [88 (25.8%) cases; 58 (17.0%) with other conditions (e.g., cord hyper helix/torsion and short cord)], mother [34 (10.0%) cases; 16 (4.7%) with hypertensive diseases in pregnancy and eight (2.3%) with diabetes], placenta [31 (9.1%) cases; 18 (5.3%) with placental abruption and eight (2.3%) with placental insufficiency], and intrapartum [28 (8.2%) cases; 25 (7.3%) with asphyxia and three (0.9%) with birth trauma]. Only eight (2.3%), three (0.9%), and two (0.6%) stillbirths were attributed to amniotic fluid, trauma and uterus, respectively.

**Table 5:** Distribution of stillbirth causes in different gestational weeks.

Causes	Gestational weeks				Total (N = 341)	*P* value*
	28–31 + 6 (N = 152)	32–33 + 6 (N = 54)	34–36 + 6 (N = 78)	37–42 (N = 57)		
***A-Fetus***	66/152 (43.4%)	18/54 (33.3%)	24/78 (30.8%)	9/57 (15.8%)	117/341 (34.3%)	0.002
A1-Lethal congenital anomaly	19/152 (12.5%)	5/54 (9.3%)	11/78 (14.1%)	7/57 (12.3%)	42/341 (12.3%)	0.874
A2-Infection	5/152 (3.3%)	0	0	0	5/341 (1.5%)	
A3-Non-immune hydrops	5/152 (3.3%)	2/54 (3.7%)	4/78 (5.1%)	1/57 (1.8%)	12/341 (3.5%)	
A4-Iso-immunization	0	0	0	0	0	
A5-Fetomaternal haemorrhage	0	0	0	0	0	
A6-Twin-twin transfusion	6/152 (3.9%)	1/54 (1.8%)	0	0	7/341 (2.1%)	
A7-Fetal growth restriction	31/152 (20.4%)	10/54 (18.5%)	9/78 (11.5%)	1/57 (1.8%)	51/341 (15.0%)	0.005
***B-Umbilical Cord***	27/152 (17.8%)	15/54 (27.8%)	19/78 (24.4%)	27/57 (47.4%)	88/341 (25.8%)	0.000
B1-Prolapse	1/152 (0.7%)	0	0	0	1/341 (0.3%)	
B2-Constricting loop or knot	6/152 (3.9%)	3/54 (5.6%)	7/78 (9.0%)	9/57 (15.8%)	25/341 (7.3%)	
B3-Velamentous	0	3/54 (5.6%)	1/78 (1.3%)	0	4/341 (1.2%)	
B4-Umbilical cord-Other	20/152 (13.2%)	9/54 (16.7%)	11/78 (14.1%)	18/57 (31.6%)	58/341 (17.0%)	0.013
***C-Placenta***	16/152 (10.5%)	2/54 (3.7%)	8/78 (10.3%)	5/57 (8.8%)	31/341 (9.1%)	0.517
C1-Placenta abruption	8/152 (5.3%)	1/54 (1.9%)	5/78 (6.4%)	4/57 (7.0%)	18/341 (5.3%)	
C2-Placenta praevia	2/152 (1.3%)	1/54 (1.9%)	0	0	3/341 (0.9%)	
C3-Vasa praevia	0	0	0	0	0	
C4-Placenta insufficiency	6/152 (3.9%)	0	1/78 (1.3%)	1/57 (1.8%)	8/341 (2.3%)	
C5-Placenta-Other	0	0	2/78 (2.6%)	0	2/341 (0.6%)	
***D-Amniotic fluid***	3/152 (2.0%)	3/54 (5.6%)	1/78 (1.3%)	1/57 (1.8%)	8/341 (2.3%)	
D1-Chorioamnionitis	2/152 (1.3%)	3/54 (5.6%)	1/78 (1.3%)	1/57 (1.8%)	7/341 (2.1%)	
D2-Oligohydramnios	1/152 (0.7%)	0	0	0	1/341 (0.3%)	
D3-Polyhydramnios	0	0	0	0	0	
D4-Amniotic fluid-Other	0	0	0	0	0	
***E-Uterus***	1/152 (0.7%)	0	1/78 (1.3%)	0	2/341 (0.6%)	
E1-Uterine rupture	1/152 (0.7%)	0	1/78 (1.3%)	0	2/341 (0.6%)	
E2-Anomalies	0	0	0	0	0	
E3-Uterus-Other	0	0	0	0	0	
***F-Mother***	18/152 (11.8%)	6/54 (11.1%)	8/78 (10.3%)	2/57 (3.5%)	34/341 (10.0%)	0.344
F1-Diabetes	5/152 (3.3%)	1/54 (1.9%)	1/78 (1.3%)	1/57 (1.8%)	8/341 (2.3%)	
F2-Thyroid abnormality	0	0	0	0	0	
F3-Chronic hypertensive disorder	4/152 (2.6%)	0	0	0	4/341 (1.2%)	
F4-Hypertensive diseases in pregnancy	7/152 (4.6%)	3/54 (5.6%)	5/78 (6.4%)	1/57 (1.8%)	16/341 (4.7%)	
F5-SLE	0	1/54 (1.9%)	0	0	1/341 (0.3%)	
F6-Cholestasis	1/152 (0.7%)	1/54 (1.9%)	2/78 (2.6%)	0	4/341 (1.2%)	
F7-Drug misuse	0	0	0	0	0	
F8-Maternal-Other	1/152 (0.7%)	0	0	0	1/341 (0.3)	
***G-Intrapartum***	12/152 (7.9%)	4/54 (7.4%)	5/78 (6.4%)	7/57 (12.3%)	28/341 (8.2%)	0.647
G1-Asphyxia	10/152 (6.6%)	3/54 (5.6%)	5/78 (6.4%)	7/57 (12.3%)	25/341 (7.3%)	0.470
G2-Birth trauma	2/152 (1.3%)	1/54 (1.9%)	0	0	3/341 (0.9%)	
***H-Trauma***	1/152 (0.7%)	1/54 (1.9%)	1/78 (1.3%)	0	3/341 (0.9%)	
H1-External trauma	0	0	0	0	0	
H2-Iatrogenic injury	1/152 (0.7%)	1/54 (1.9%)	1/78 (1.3%)	0	3/341 (0.9%)	
***I-Unclassified***	8/152 (5.3%)	5/54 (9.3%)	11/78 (14.1%)	6/57 (10.5)	30/341 (8.8%)	0.149
I1-No relevant condition identified	8/152 (5.3%)	5/54 (9.3%)	11/78 (14.1%)	6/57 (10.5%)	30/341 (8.8%)	
I2-No information available	0	0	0	0	0	

Data are n/N (%) for cases. The criteria used for categorization are based on the ReCoDe classification system.

*Comparison of different gestational weeks.

The 51 cases of FGR comprised 35 cases of singleton FGR, eight cases of SIUGR (selective intrauterine growth restriction), and eight cases of DCDA (dichorionic diamniotic). Among the B-umbilical cord causes, B4 (umbilical cord-other) were most common [58 (17.0%) cases], comprising 50 cases of cord hyper helix/torsion, three of short umbilical cord, two of single umbilical artery, one of cord entangled by the amniotic band, one of cord atresia, and one case of cord cyst. Of the intrapartum causes, 25 stillbirths were attributed to acute asphyxia because of sudden rupture of cord vessels (four cases), hypertensive diseases in pregnancy (five cases), lethal congenital anomaly (four cases), cord prolapse (three cases), FGR (three cases), placental abruption (two cases), placental insufficiency (two cases), hyper helix of the umbilical cord (one case), and non-immune hydrops (one case).

### Distribution of stillbirth causes in different gestational weeks

Most of the third-trimester stillbirths caused by fetal conditions occurred at 28–31 + 6 weeks of gestation, accounting for 43.4% (66/152), which was significant (P = 0.002). Most third-trimester stillbirths caused by umbilical cord occurred at 37–42 weeks of gestation, accounting for 47.4% (27/57), which was also significant (P = 0.000). The lowest frequency of third-trimester stillbirths caused by maternal conditions was at 37–42 weeks of gestation, accounting for 3.5% (2/57), but this was not significant. Similar results were obtained for other factors. The lowest frequency of third-trimester stillbirths caused by placental factors occurred at 32–33 + 6 weeks of gestation, accounting for 3.7% (2/54), and a trend toward a higher incidence of third-trimester stillbirths of intrapartum causes at 37–42 weeks of gestational age was observed, accounting for 12.3% (7/57), but this was not significant (Table 5).

## Discussion

The incidence of stillbirth in the Women’s Hospital, Zhejiang University School of Medicine from January 1, 2013, to December 31, 2018, was 3.06/1000 (2.63–3.63/1000), lower than the national rate in previous reports^5,6^. Stillbirth was considered in relation to maternal age, education level, social status, and antenatal care of pregnant women^13,14^. We found that 78.8% of mothers were of an appropriate age (20–34 years) and 58.6% had a college or postgraduate degree. Furthermore, 13.0% of the pregnant women did not have regular antenatal care, and 4.1% of the mothers with stillbirth were engaged in physical labor. The mature obstetric techniques and extensive experience of prenatal care in our hospital may explain the low rate of stillbirths.

Collecting high-quality data and applying a reliable classification system constitute the first step in understanding the causes of stillbirth. Our understanding of stillbirth causes in LMICs is based on analysis of registration data, not investigation of the placenta or autopsy of the fetus. A systematic review of stillbirths from 2009 to 2016 highlighted the poor-quality data to determine the cause of stillbirths^10^. Although placental examination is highly informative, it was not performed in studies from low-income settings, and in only two small-scale studies in middle-income settings. An investigation of 2847 stillbirths in seven low-income countries using a computer-based hierarchal algorithm based on medical records^15^ and did not include a biological investigation of the placenta or autopsy of the fetus. Notably, the underlying causes of stillbirths were not fully characterized because biological investigations were not conducted. In this retrospective study, we reviewed available maternal and fetal medical records during pregnancy and delivery, and undertook macroscopic and histological examination of the placenta, and autopsy and array chip testing of the fetus. Unfortunately, only 64 of 341 stillbirths were subjected to placental examination. The recommended fetal autopsy rate is > 75%. However, the reported autopsy rate is 5.8–58% for various reasons, including religion, cognition of stillbirths, and the educational level of pregnant women^16^. In this study, only 36 autopsy cases (10.6%) were reported. In addition, two abnormal results were found among 11 stillbirths subjected to array chip testing, but the abnormality was not directly related to the clinical phenotype. The cause of stillbirth in one case was classified as B2 (constricting loop of umbilical cord), and the other as I1 (no relevant condition identified).

The aforementioned systematic review of stillbirths published in 2018 also highlighted the inconsistent use of classification systems for causes of stillbirths. A variety of classification systems are available^17,18^. The decline in the stillbirth rate in high-income countries has slowed or stopped during the last few decades after decreasing by two-thirds from 1950 to 1975^19^. Standardization of classification systems is required to improve our understanding of stillbirth. In 2011 the authors of the Lancet Stillbirths Series suggested that classification should be the primary focus of epidemiological measurement research^20^. For each stillbirth, many possible causative conditions are present, and integrating the information is challenging. The ReCoDe classification organizes clinical information related to stillbirth rather than the cause of death^21^. The purpose of ReCoDe is to avoid a case-by-case analysis and it enables retrospective classification using databases. Although ReCoDe is less clinically effective than a case-by-case perinatal review^22^, it is less time-consuming, can be performed retrospectively, and accounts for inconsistencies among researchers, countries, and periods. Another advantage of the ReCoDe system is its ICD-code-based structural hierarchy, resulting in 85% of stillbirth cases meeting relevant conditions. However, it follows a pre-established hierarchy, irrespective of whether another condition made a greater contribution. In 2009, among the six stillbirth classification systems evaluated by the International Stillbirth Alliance^23^, RECODE ranked third. The optimum classification system should collect as much relevant information as possible, using a hierarchical method as a guide but still rely on expert advice^24^.

We evaluated the RECODE classification in a retrospective analysis of third-trimester stillbirths. The rate of unexplained cases (30/341, 8.8%) was slightly lower than that in a West Midlands cohort of 2625 stillbirths, an Italian sample of 154 stillbirths, a Dutch sample of 485 antepartum singleton stillbirths, and a French sample of 969 stillbirths (16.0, 14.3, 14.2, and 13.9%, respectively)^20,21,25,26^. We reported a 12.3% (42/341) rate of lethal congenital anomalies, similar to the finding of Gardosi, but the rate of stillbirths classified as FGR (A7) was significantly lower (15.0% versus 43.0%). In the three other case series, the rate of FGR was 30.3%, 16.9%, and 38.2%^20,25,26^. The differences may be a result of population enrollment and different definitions of FGR. The rate of FGR stillbirths may have been slightly underestimated in this study.

FGR is a common obstetrics complication. It is typically associated with adverse outcomes such as premature birth, stillbirth, and neonatal death. The incidence in the United States and Europe is 5–15%^27^, while that in China is similar to that of developed countries. Of the above-mentioned six stillbirth classification systems, only the ReCoDe system classifies FGR separately^23^. Of the 341 cases of third-trimester stillbirths, there were 97 cases of FGR in single fetuses [32.6% (97/298)], and 27 cases of FGR in twin fetuses [62.3% (27/43)]. Among them, 73 stillbirths had other conditions that were greater contributors than FGR, such as maternal status (including F4-severe pre-eclampsia, F1-gestational diabetes with poor glycemic control), fetal status (including A1-fetal congenital anomaly, A3-fetal non-immune hydrops), placental status (including C4-severe placental insufficiency, C1-placental abruption), umbilical factors (including B1-umbilical cord prolapse, B4-umbilical vascular rupture) and intrapartum (G), while the remaining 51 cases were classified as fetal growth restriction (A7). Termination of pregnancy according to the fetal condition is the mainstay of treatment for FGR. FGR pregnant women with abnormal doppler measurement of blood flow in the ductus venosus (DV) have a significantly increased incidence of stillbirth. The combination of fetal movement and ultrasound monitoring of blood flow parameters can reduce the incidence of FGR^28^.

A meta-analysis concluded that stillbirths in low-income countries were attributable to infection (15.8%), hypoxic peripartum death (11.6%), antepartum hemorrhage (9.5%), and other unspecified conditions (13.8%); 41–44% were unexplained^10^. In a study in seven low-income countries^15^, the main causes of stillbirth were asphyxia (46.6%), infection (20.8%), congenital anomalies (8.4%), prematurity (6.6%), and unidentified (11.8%). Infection was the leading cause of stillbirth in all of the above studies. In this work, the rate of chorioamnionitis (D1) was 2.1% (7/341), significantly lower than reported by others. The differences could be caused by inconsistent use of classification systems. In particular, the overall incidence of stillbirths in China has reached the level of high-income countries, and so the classification of causes of stillbirths has become similar to that in high-income countries. Among the seven cases of chorioamnionitis, two were clinically diagnosed, and the other five had pathological findings upon placental examination. In this study, among 64 cases in which placental pathology was evaluated, 26 cases of chorioamnionitis were histologically confirmed. Excluding the five cases mentioned above (D1), those 21 cases were classified as follows: 10 cases as umbilical cord-other (B4), two as constricting loop of umbilical cord (B2), two as lethal congenital anomaly (A1), two as hypertensive diseases in pregnancy (F4), two as diabetes (F1), one as placental insufficiency (C4), one as infection of fetus (A2) and one as intrapartum asphyxia (G1).

Although this study was conducted in an HBV-endemic area, no stillbirth was attributed to HBV. The incidence of third-trimester stillbirths was similar in HBV-negative (0.317%, 317/99,879) and HBV-positive (0.289%, 21/7263) pregnant women. Furthermore, five women were seropositive for syphilis, and four stillbirths were attributed to syphilis (A2-infection of fetus); the other was attributed to other conditions (A7-fetal growth restriction). The four syphilis-seropositive women classified as A2 (infection of fetus) did not attend routine antenatal care in our hospital. Three of them had stillbirths at the time of diagnosis of syphilis, and one had been treated with benzylpenicillin for 4 weeks in another hospital but had a titer of 1:16. The syphilis-seropositive woman classified as A7 (fetal growth restriction) was treated with two courses of benzylpenicillin in a foreign hospital for 5 weeks, and had a titer of 1:1. Otherwise, the incidence of third-trimester stillbirth in syphilis-seropositive pregnant women (2.793%, 5/179) was significantly higher than that in syphilis-seronegative pregnant women (0.311%, 333/106,963). This emphasizes the importance of syphilis screening and treatment.

The above-mentioned studies reported a 46.6% rate of asphyxia and 11.6% of hypoxic peripartum death^10,15^, an important cause of stillbirth. Abnormal conditions of the mother, placenta, and umbilical cord (*e.g.*, preeclampsia, placental abruption, and umbilical cord prolapse) may cause fetal hypoxia, asphyxia, and death. In this study, the rate of asphyxia (G1) was 7.3% (25/341), significantly lower than reported previously. In the ReCoDe classification system, asphyxia is in the G1 subcategory of the G category (intrapartum). Among the 28 (8.2%) cases of G category (intrapartum), 25 stillbirths were attributed to acute asphyxia (G1) and the other three to birth trauma (G2), all of which were breech deliveries prior to 34 weeks. In fact, other conditions made greater contributions to stillbirths in these three cases of birth trauma but were classified as G2 because the direct cause was breech delivery. The same is true of 25 cases of acute asphyxia (G1), which were classified as sudden rupture of cord vessels (four cases), hypertensive diseases in pregnancy (five cases), lethal congenital anomaly (four cases), cord prolapse (three cases), FGR (three cases), placenta abruption (two cases), placenta insufficiency (two cases), hyper helix of umbilical cord (one case), and non-immune hydrops (one case). Early detection and treatment of complications during pregnancy (e.g., preeclampsia, FGR), enhanced fetal monitoring during birth (fetal heart rate monitoring, ultrasound monitoring), and rational use of midwifery technology (cesarean section, forceps, head-up attraction, perineal side cut), can reduce the incidence of intrapartum stillbirths by 45%^29^.

Placental investigation showed that 9.1% (31/341) of stillbirths were attributed to placenta (C), and 2.3% (8/341) to placental insufficiency (C4). The Stillbirth Collaborative Research Network study^11^ showed that placental examination can diagnose 64.6% of stillbirths. A prospective, observational study in South Africa^30^ involved macroscopic and histological examination of the placenta and culture of fetal blood. These examinations should be considered for future studies in LMIC settings. Our findings highlight the need for systematic investigation of stillbirth, including investigation of the placenta and medical records.

This study had several limitations. First, we did not use methods of determining the causes of stillbirth, such as antiphospholipid antibody testing (useful in 1.1% of cases), fetal-maternal hemorrhage testing (6.4%), and testing for bacteria (such as group B *Streptococcus*)^31^. We tested a small number of women for viruses (such as parvovirus B), which have been implicated as causes of stillbirths^32^. In addition, a complete autopsy (36/341) and karyotyping (11/341) were performed in a few cases, although those enabled identification of the cause of stillbirth in 42.3% and 11.9% of cases. The limited placental investigation may attribute to limited placental cause. Second, we retrospectively abstracted maternal and fetal clinical information from available medical records. Therefore, incomplete medical records may have contributed to unclassified (I) stillbirths (8.8%). Third, this was a single-center study. Although our research center is the largest in Hangzhou, cooperation with other research centers would make our findings more generalizable. Fourth, a larger control group may have been better.

Targeted investigation can ascertain the causes of most third-trimester stillbirths, reducing the incidence of stillbirths globally. We performed this study to obtain reliable medical evidence that could be used to reduce the rate of preventable stillbirths. Although our findings provide important insight into the causes of third-trimester stillbirth, the results may not be generalizable to other resource-poor areas. For example, in areas where interventions such as caesarean section are difficult to obtain, the rate of intrapartum causes of stillbirth may be higher. Further studies in diverse LMIC settings are needed to improve our understanding of the strategies, interventions, and research priorities necessary for achieving the goals of the ‘Every Newborn Action Plan’ by 2030.

## Methods

### Definition of stillbirth

The gestational weeks definition of stillbirth varies among countries and is ≥ 20 gestational weeks in high-income countries because of their advanced neonatal care^2^. The World Health Organization (WHO) defines stillbirth as fetal death at ≥ 28 weeks of gestation, 1000 g birth weight, or 35 cm birth length—the International Classification of Diseases definitions of third-trimester stillbirth^33^. Several factors affect the incidence of stillbirths, and the risk factors vary according to gestational weeks. In this study, we defined third-trimester stillbirths as fetal death at ≥ 28 gestational weeks.

### Procedures

This retrospective study was conducted at the Women’s Hospital, Zhejiang University School of Medicine, where 111,275 perinatal fetuses were delivered from January 1, 2013, to December 31, 2018. We recruited only fetuses with a gestational age of at least 28 weeks. Gestational age staging was based on the last menstrual period date and obstetric ultrasound in the first trimester. Excluding those without complete information, a total of 338 women who had third-trimester stillbirths were enrolled, and 291 women with live births formed the control group, which were randomly selected from within the same period as the stillbirths, matched on date.

The maternal and fetal clinical information of the cases was abstracted from the medical records. The information was collected by a member of the study staff and was reviewed by a physician researcher using a standard data-collection form.

The information collected included: (1) General information: age of delivery, nationality, education level, domicile, stable occupation, weight, height, and body mass index of pregnant women; (2) antenatal care visit, gestational weeks, parity, mode of conception, multiple gestation, chorionic properties of twins, history of adverse contact in early pregnancy, history of preventing miscarriage; (3) history of maternal diseases, history of uterine surgery, complications of previous pregnancy, complications of current pregnancy; (4) previous inevitable abortion, spontaneous abortion, recurrent abortion or stillbirth; (5) genetic or structural abnormality of the fetus, fetal growth restriction (FGR); (6) pathological conditions of the umbilical cord; (7) pathological conditions of the placenta; (8) laboratory indicators: TORCH virus, parvovirus, genital tract pathogens, prenatal screening, ABO blood group, Rh blood group, maternal syphilis, hepatitis B virus (HBV) and human immunodeficiency virus test, alpha-fetoprotein, oral glucose tolerance test, glycated hemoglobin, thyroid function, triglyceride, total cholesterol, glycocholic acid, total bile acids, hemoglobin, coagulation function; (9) clinical manifestations; (10) timing of stillbirth (antepartum/intrapartum); (11) delivery conditions: mode of delivery, neonatal weight, neonatal sex, amniotic fluid, neonatal appearance, macroscopic observation of umbilical cord and placenta; (12) macroscopic observation of umbilical cord and placenta, array test, and autopsy; and (13) maternal adverse outcomes.

The study was approved by the Human Research Ethics Committee of the Women’s Hospital, Zhejiang University School of Medicine. The Human Research Ethics Committee agreed that this study is exempt from informed consent because there will be no additional adverse effects on participants, and the investigator will strictly observe the principle of confidentiality, and the relevant study information will only be accessible to the investigator. The methods were performed in accordance with the relevant guidelines and regulations.

### Laboratory procedures

The placenta was retrieved and examined macroscopically after delivery. The placenta, membranes, and umbilical cord were immersed in 10% buffered formalin and transported to the Pathology Department of the Women’s Hospital, Zhejiang University School of Medicine. Portions of placenta selected by the histopathologist were embedded in paraffin and processed for routine hematoxylin and eosin staining, using the standard protocols for placental and umbilical cord assessment.

After delivery, a strip of muscle tissue (1 cm) was resected from the thigh and placed in a sterile container for testing using the CytoScan HD Array Chip (Affymetrix Company) by the Department of Reproductive Genetics of our hospital. The CytoScan HD Array Chip contains 75,000 single nucleotide polymorphism markers and 19,000 copy number variation markers. They focus on chromosomal microdeletions, duplications, and abnormalities, as well as subtendyme deletions, of known clinical significance.

After delivery, the dead fetus was transported to the Pathology Department for autopsy for assessment of general congenital defects, morphological deformities, and subtle abnormalities. Autopsy can also identify infection, anemia, hypoxia, and metabolic abnormalities.

### Determination the single cause of stillbirths

Individual cases were reviewed by at least two obstetricians and the causes of third-trimester stillbirths were categorized by the Relevant Condition at Death (ReCoDe) classification system. The ReCoDe classification includes nine categories from A (fetal conditions) to I (unclassified), each of which is divided into several subgroups, for a total of 37 subcategories. Although multiple medical conditions may be associated with third-trimester stillbirth, each third-trimester stillbirth was assigned a single cause.

Each case of third-trimester stillbirths was discussed by the expert group in our hospital based on all available information (including clinical, pathological, genetic, autopsy, etc.), confirmed the single cause and recorded.

### Statistical analysis

Categorical variables are presented as frequencies (%). The demographic and clinical features of mothers and fetuses were compared by χ^2^ test, as were risk factors for stillbirth according to number of gestational weeks. Statistical analysis was performed using SPSS 20.0 software. A value of P < 0.05 was regarded as indicative of statistical significance.

## Supplementary Information

Supplementary Information.

## Data Availability

The datasets generated during and/or analysed during the current study are available from the corresponding author on reasonable request.
