# Supplementary material for: Risk factors and incidence of third trimester stillbirths in China
Source: Sci Rep. 2021 Jun 16;11:12701. doi: 10.1038/s41598-021-92106-1 (PMC8209053; doi:10.1038/s41598-021-92106-1)
Supplement: Supplementary file 1 — Supplementary Information. [file 41598_2021_92106_MOESM1_ESM.pdf]

Risk Factors and Incidence of Third Trimester Stillbirths in China

Ying Hu[1#], Qi Wu[1#], Jia Liu[1], Die Hong[1], Yuqing Zou[1], Jingjing Lu[1], Yuhui Wang[1], Danqing Chen[1], Lu Qi[2*], Zhaoxia Liang[1,2*]

[1] Obstetrical Department, Women's Hospital, School of Medicine, Zhejiang University, Hangzhou, China

[2] Department of Epidemiology, School of Public Health and Tropical Medicine, Tulane University, New Orleans, LA

[#] Ying Hu and Qi Wu contributed equally to this work

[*] Co-corresponding author

[*]**Correspondence to** Zhaoxia Liang. Obstetrical Department, Women's Hospital, School of Medicine, Zhejiang University, Hangzhou, China. 310006. E-mail: xiaozaizai@zju.edu.cn, Tel: +86571-87061501

Supplement: Incidence of third-trimester stillbirths in women and fetuses with different demographic features

Among the total 107142 mothers, the incidence of third-trimester stillbirths of resident household registration was 0.241% (86/35642), while that of non-resident household registration was 0.352% (252/71500). The highest incidence of third-trimester stillbirths was found in mothers younger than 20 years old, which was 2.381% （4/168）, while the incidence of other age groups was 0.349% （165/47331） between 20 and 29 years old, 0.279% （108/38672） between 30 and 34 years old, and 0.291% (61/20971) in over 35 years old.

The incidence of third-trimester stillbirths was 0.315% (315/99879) in HBV-negative pregnant women and 0.317% (23/7263) in HBV-positive pregnant women. Among syphilis seronegative pregnant women, the incidence of third-trimester stillbirths was 0.311% (333/106963), while in syphilis seropositive pregnant women, the incidence increased significantly to 2.793% (5/179).

In single pregnant women, the incidence of third-trimester stillbirths was 0.289% （298/103058）, while in twin pregnant women, the incidence increased significantly to 0.991% （40/4035）, of which MCMA accounted for 2.5% （1/40）, MCDA 50.0% （20/40）, DCDA 42.5% （17/40）, and two cases of unknown chorionic property. Interestingly, the incidence of third-trimester stillbirths was 0 in triplets' pregnant women.

The overall incidence of third-trimester stillbirths in female fetuses （0.298%， 159/53420）is slightly higher than that in male fetuses （0.265%， 153/57819）,excluding 29 with unknown sex.

Table S1. Incidence of third-trimester stillbirths in women and fetuses with different demographic features

	mothers of perinatal fetuses（N）	mothers of third-trimester stillbirths	
		n	incidence（%）
Household registration	107142	338	
Resident	35642	86	0.241
Non-resident	71500	252	0.352
Age, years			
<20	168	4	2.381
20-29	47331	165	0.349
30-34	38672	108	0.279
≥35	20971	61	0.291
Hepatitis B			
negative	99879	315	0.315
positive	7263	23	0.317
syphilis			
negative	106963	333	0.311
positive	179	5	2.793
Multiple gestation			
singleton	103058	298	0.289
twins	4035	40	0.991
MCMA		1	2.5
MCDA		20	50.0
DCDA		17	42.5
unknown chorionic property		2	5.0
triplet	49	0	0
infant sex			

male	57819	153	0.265
female	53420	159	0.298
unknown	36	29	80.6

Data are N, n and n/N (%) for cases.
